# *Commiphora myrrha* n-hexane extract suppressed breast cancer progression through induction of G0/G1 phase arrest and apoptotic cell death by inhibiting the Cyclin D1/CDK4-Rb signaling pathway

**DOI:** 10.3389/fphar.2024.1425157

**Published:** 2024-08-05

**Authors:** Huiming Huang, Jinxin Xie, Fei Wang, Shungang Jiao, Xingxing Li, Longyan Wang, Dongxiao Liu, Chaochao Wang, Xuejiao Wei, Peng Tan, Pengfei Tu, Jun Li, Zhongdong Hu

**Affiliations:** ^1^ School of Chinese Materia Medica, Beijing University of Chinese Medicine, Beijing, China; ^2^ Modern Research Center for Traditional Chinese Medicine, Beijing Research Institute of Chinese Medicine, Beijing University of Chinese Medicine, Beijing, China; ^3^ Dongfang Hospital, Beijing University of Chinese Medicine, Beijing, China

**Keywords:** CMHE, breast cancer, G0/G1 phase arrest, apoptosis, Cyclin D1/CDK4-Rb signaling pathway

## Abstract

**Background:**

Breast cancer (BC) is one of the most frequently observed malignancies globally, yet drug development for BC has been encountering escalating challenges. *Commiphora myrrha* is derived from the dried resin of *C. myrrha* (T. Nees) Engl., and is widely adopted in China for treating BC. However, the anti-BC effect and underlying mechanism of *C. myrrha* remain largely unclear.

**Methods:**

MTT assay, EdU assay, and colony formation were used to determine the effect of *C. myrrha* n-hexane extract (CMHE) on the proliferation of human BC cells. Cell cycle distribution and apoptosis were assessed via flow cytometry analysis. Moreover, metastatic potential was evaluated using wound-scratch assay and matrigel invasion assay. The 4T1 breast cancer-bearing mouse model was established to evaluate the anti-BC efficacy of CMHE *in vivo*. RNA-sequencing analysis, quantitative real-time PCR, immunoblotting, immunohistochemical analysis, RNA interference assay, and database analysis were conducted to uncover the underlying mechanism of the anti-BC effect of CMHE.

**Results:**

We demonstrated the significant inhibition in the proliferative capability of BC cell lines MDA-MB-231 and MCF-7 by CMHE. Moreover, CMHE-induced G0/G1 phase arrest and apoptosis of the above two BC cell lines were also observed. CMHE dramatically repressed the metastatic potential of these two cells *in vitro*. Additionally, the administration of CMHE remarkably suppressed tumor growth in 4T1 tumor–bearing mice. No obvious toxic or side effects of CMHE administration in mice were noted. Furthermore, immunohistochemical (IHC) analysis demonstrated that CMHE treatment inhibited the proliferative and metastatic abilities of cancer cells, while also promoting apoptosis in the tumor tissues of mice. Based on RNA sequencing analysis, quantitative real-time PCR, immunoblotting, and IHC assay, the administration of CMHE downregulated Cyclin D1/CDK4-Rb signaling pathway in BC. Furthermore, RNA interference assay and database analysis showed that downregulated Cyclin D1/CDK4 signaling cascade participated in the anti-BC activity of CMHE.

**Conclusion:**

CMHE treatment resulted in the suppression of BC cell growth through the stimulation of cell cycle arrest at the G0/G1 phase and the induction of apoptotic cell death via the inhibition of the Cyclin D1/CDK4-Rb pathway, thereby enhancing the anti-BC effect of CMHE. CMHE has potential anti-BC effects, particularly in those harboring aberrant activation of Cyclin D1/CDK4-Rb signaling.

## Graphical Abstract

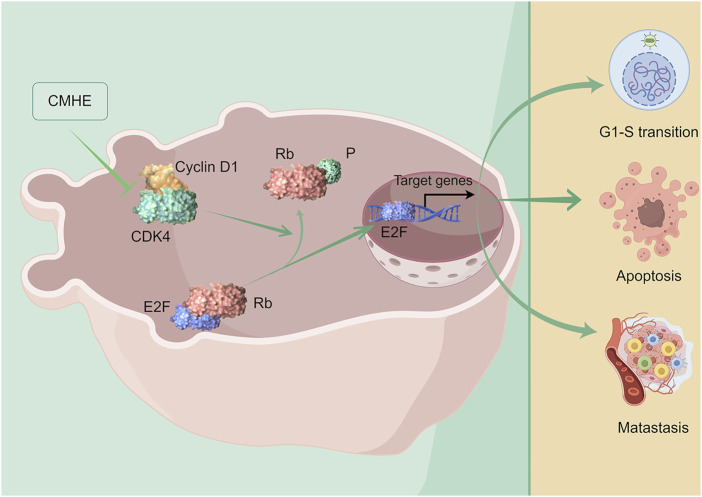

## 1 Introduction

Breast cancer (BC) is among the main health concerns among women. Since BC has no typical symptoms in its early stages, it is not easily detectable (Accordino et al., 2016). In advanced stages, BC often metastasizes, leading to organ failure and life-threatening events in patients (Hortobagyi et al., 2017). Current treatments for BC include surgery, endocrine therapy, targeted therapy, chemotherapy, and radiotherapy (Tarantino et al., 2022). Recently, traditional Chinese medicine (TCM) has become increasingly important in treating cancer with its unique advantages. Furthermore, research on the anti-BC effects of TCM has garnered increasing attention from researchers and seems to have quite a broad prospect (Hung et al., 2022; Peng et al., 2022).

Xihuang Pill is composed of *Bovis Calculus*, *Moschus*, Frankincense, and *Commiphora myrrha*, which is one of the fourteen nationally approved anti-cancer Chinese patent medicines and has been extensively used in clinical treatment of BC in China (Wu et al., 2020; Ge et al., 2022). *Commiphora myrrha* is derived from the dried resin of *C. myrrha* (T.Nees) Engl. *C. myrrha* is flat in nature, pungent, and bitter in taste, and has the effects of promoting blood circulation, removing blood stasis, detumescence, and promoting granulation, which is often used clinically in the treatment of BC (Shen et al., 2012; Xu et al., 2023). Modern pharmacological studies have depicted the antibacterial, anti-inflammatory, antitumor, hepatoprotective, analgesic, neuroprotective, and hypolipidemic activities of *C. myrrha* extract and its monomeric compounds (Shen et al., 2012; Madia et al., 2021). Nevertheless, the underlying mechanisms of the anti-BC effect of *C. myrrha* remain greatly unknown.

The results revealed the dramatic repression of the growth of human BC cell lines (MDA-MB-231 and MCF-7) by *C. myrrha* n-hexane extract (CMHE). Moreover, CMHE promoted apoptosis and induced cell cycle arrest at the G0/G1 phase in these two cells. CMHE also markedly repressed BC cell invasion and migration. In addition, the administration of CMHE significantly blocked tumor development in the 4T1 tumor-bearing mice. Intriguingly, the downregulation of the Cyclin D1/CDK4-Rb signaling cascade was associated with the anti-BC effect of CMHE. Therefore, CMHE is a potential anti-BC therapeutic agent, particularly for those harboring aberrant activation of Cyclin D1/CDK4-Rb signaling.

## 2 Materials and methods

### 2.1 Reagent and antibodies

Reagents including DMEM, 0.25% trypsin, FBS, RPMI-1640, and penicillin-streptomycin were acquired from Corning Life Sciences (Corning, NY, United States). Lipofectamine 2000 and Opti-MEM medium were acquired from Invitrogen (Invitrogen, Carlsbad, CA). β-actin (sc-47778) antibody was obtained from Santa Cruz Biotechnology (Santa Cruz, CA, United States). Cyclin D1 (55506T), CDK4 (12790T), Rb (9309T), and p-Rb (8516T) antibodies were procured from Cell Signaling Technology Corporation (Danvers, MA, United States) and the chemiluminescent HRP substrate (ECL) was provided by Millipore Corporation (Billerica, MA, United States).

### 2.2 Preparation of CMHE

*Commiphora myrrha* was procured from Beijing Huamiao Pharmaceutical Co. Ltd. (Kenya, DD4271). Around 3.0 kg of *C. myrrha* was crushed and extracted with n-hexane at reflux for 2 h. After filtrate combination, solvent recovery at reduced pressure and freeze-drying, 651.0 g CMHE was obtained. The chemical composition analysis of CMHE was carried out (Supplementary Figure S1). The previous study has revealed that the chemical components of CMHE primarily comprise sesquiterpenoids, diterpenoids, triterpenoids, dimeric sesquiterpenoids, steroids, lignans, and flavonoids (Wang et al., 2022). The stock concentration of CMHE in DMSO is 40 mg/mL.

### 2.3 Cell culture

The human BC cell lines (MDA-MB-231 and MCF-7) were acquired from the Cell Resource Center, Institute of Basic Medical Sciences, Chinese Academy of Medical Sciences (Beijing, China). In addition, cells were cultured using RPMI-1640 or DMEM covering 1% penicillin-streptomycin and 10% FBS at 37°C with 5% CO_2_, separately.

### 2.4 Cell viability assay

Cells (2.5 × 10^3^/well) were inoculated into 96-well plates and were subjected to treatment with CMHE (0, 10, 15, 20, 25, and 30 μg/mL) for 24/48/72 h, separately. Subsequently, 10% MTT solution (0.5 mg/mL, 100 µL) was introduced in each well. After 4-h of incubation at 37°C, 150 µL DMSO was supplemented to replace the MTT solution in the 96-well plates. The 96-well plate was later shaken for 5–10 min and finally, the optical density (OD) values were measured at 490 nm with the use of the microplate reader.

### 2.5 EdU cell proliferation assay

In this assay, cells were inoculated into 12-well plates, followed by 24 h of incubation until adherence and 48 h of CMHE treatment. Cell proliferation was then detected using the BeyoClick™ EdU-488 kit (C0071, Biyuntian Biotechnology, Shanghai, China) as per the instructions of the manufacturer. Cell imaging was carried out using an inverted fluorescence microscope (DMIL LED, Leica, Germany).

### 2.6 Colony formation assay

This assay involved the inoculation of cells for 12 days in a 10 cm dish (2.5 × 10^3^ cells/dish) before CMHE treatment. After rinsing with PBS thrice, cells were fixed with 4% paraformaldehyde (PFA) solution under ambient temperature for a 15-min period. Finally, 0.1% crystal violet solution was used for 30 min to accomplish cell staining.

### 2.7 Cell cycle detection

Cells were inoculated into 6-well plates, followed by 24 h of incubation until adherence. After starving the cells for 12 h, CMHE was added for a 48 h period, followed by FACA with the BD Propidium Iodide/Ribonuclease Staining Solution kit (550825, BD Biosciences Pharmingen, US) as per the manufacturers’ instructions.

### 2.8 Apoptosis analysis

After inoculation in the 6-well plates, cells were exposed to 48 h of CMHE treatments. Subsequently, FACA was carried out with an Annexin V-FITC apoptosis assay kit (556547, BD Biosciences Pharmingen, US) following the instructions of the manufacturer.

### 2.9 Wound-scratch assay

After inoculation of the cells in 12-well plates, a wound-healing assay was conducted in accordance with the previous description (Hu et al., 2016). Cell observation and imaging were performed using the inverted fluorescence microscope at 0, 12, and 24 h, respectively.

### 2.10 Matrigel invasion assay

After 12-h of starvation, the cells were exposed to the cell invasion assay (Hu et al., 2016). Firstly, CMHE was added to treat cells for 16/24 h, separately. After staining, cell photographs were obtained using the inverted fluorescence microscope, and quantitative analysis of the cells penetrating the membrane was carried out.

### 2.11 RNA-sequencing analysis

CMHE (30 μg/mL) was added to treat MCF-7 cells for a period of 48-h, followed by the extraction of total RNA with the FastPure^®^ Cell^/^Tissue Total RNA Isolation Kit V2 (Vazyme, Nanjing, China). Illumina Hiseq2500 mRNA sequencing was conducted by Shanghai Biotechnology Corporation (Shanghai, China) and the Hisat2 and Stringtie software were used for data analysis.

### 2.12 Quantitative Real-Time PCR

CMHE (30 μg/mL) was added for 24 h to accomplish cell treatment. After extracting total RNA, qRT-PCR was carried out (Yang et al., 2019) using primers obtained from Beijing Genomics Institute (Beijing, China) with the following sequences:

Cyclin D1 Forward Primer: 5′- CCG​TCC​ATG​CGG​AAG​ATC-3’;

Cyclin D1 Reverse Primer: 5′- GAA​GAC​CTC​CTC​CTC​GCA​CT-3’.

GAPDH Forward Primer: 5′-CAG​TGC​CAG​CCT​CGT​CTC​AT-3’; GAPDH Reverse Primer: 5′-AGG​GGC​CAT​CCA​CAG​TCT​TC-3’.

### 2.13 Immunoblotting

Cell lysates were harvested from the BC cells after 48 h of CMHE treatment with lysis buffer (10% glycerol, 100 mM DTT, 10 mM Tris (pH 6.8), 2% SDS), and heated at 99°C for 10 min. Subsequently, the protein levels were examined using an immunoblotting assay (Hu et al., 2015).

### 2.14 RNA interference

Cells were seeded into 6-well plates, followed by anti-Cyclin D1 or CDK4 siRNA transfection using Lipofectamine 2000. The synthesis of siRNA oligonucleotides and sequences was carried out by GenePharma Co., Ltd. (Suzhou, China), and the sequences were as follows:

Cyclin D1: 5′-UGG​AAU​AGC​UUC​UGG​AAU​U-3’;

CDK4: 5′-CUC​UUA​UCU​ACA​UAA​GGA​U-3’;

Negative Control (NC): 5′-UUC​UCC​GAA​CGU​GUC​ACG​UTT-3’.

### 2.15 Administration of CMHE in mouse breast cancer model

A subcutaneous mouse tumor model was constructed as previously described (Sun et al., 2011). BALB/c mice (6–7 weeks, female) were acquired from the Charles River Laboratories (Beijing, China). Mice were given a subcutaneous injection of mouse BC 4T1 cells (2 × 10^6^) via the right flank. All mice were observed daily and randomized into three groups (n = 9 each) after tumor volumes were 100–200 mm^3^. The groups included mice in which 1) PBS was intragastrically administered (i.g.), once/day; 2) 300 mg/kg CMHE was intragastrically (i.g.) administered, once/day; 3) 20 mg/kg 5-fluorouracil was intraperitoneally (i.p.) injected, every other day. Tumor volume and mouse weights were monitored at an interval of 2 days, and no mortality was observed in all groups during the experimental period. The formula length multiplied by width^2^/2 was used to determine the tumor volume. All the experimental protocols in this study using animal models obtained ethical clearance from the Ethics Committee of Beijing University of Chinese Medicine (No. BUCM-4-2021031603-1072).

### 2.16 Immunohistochemical analysis

The organs and tumor tissues from 4T1 BC-bearing mice treated with CMHE were fixed with 4% PFA before being embedded in paraffin and subsequently dewaxed. Histological examination using H&E staining and IHC analysis was conducted following established protocols (Jin et al., 2017).

### 2.17 Statistical analysis

Results were represented to be mean ± SEM. Two-tailed Student *t*-test was employed to explore the differences between both groups with GraphPad Prism 8.0 software. *P* < 0.05 was thought to be of statistical significance.

## 3 Results

### 3.1 CMHE inhibited human BC cell proliferation

**FIGURE 1:**
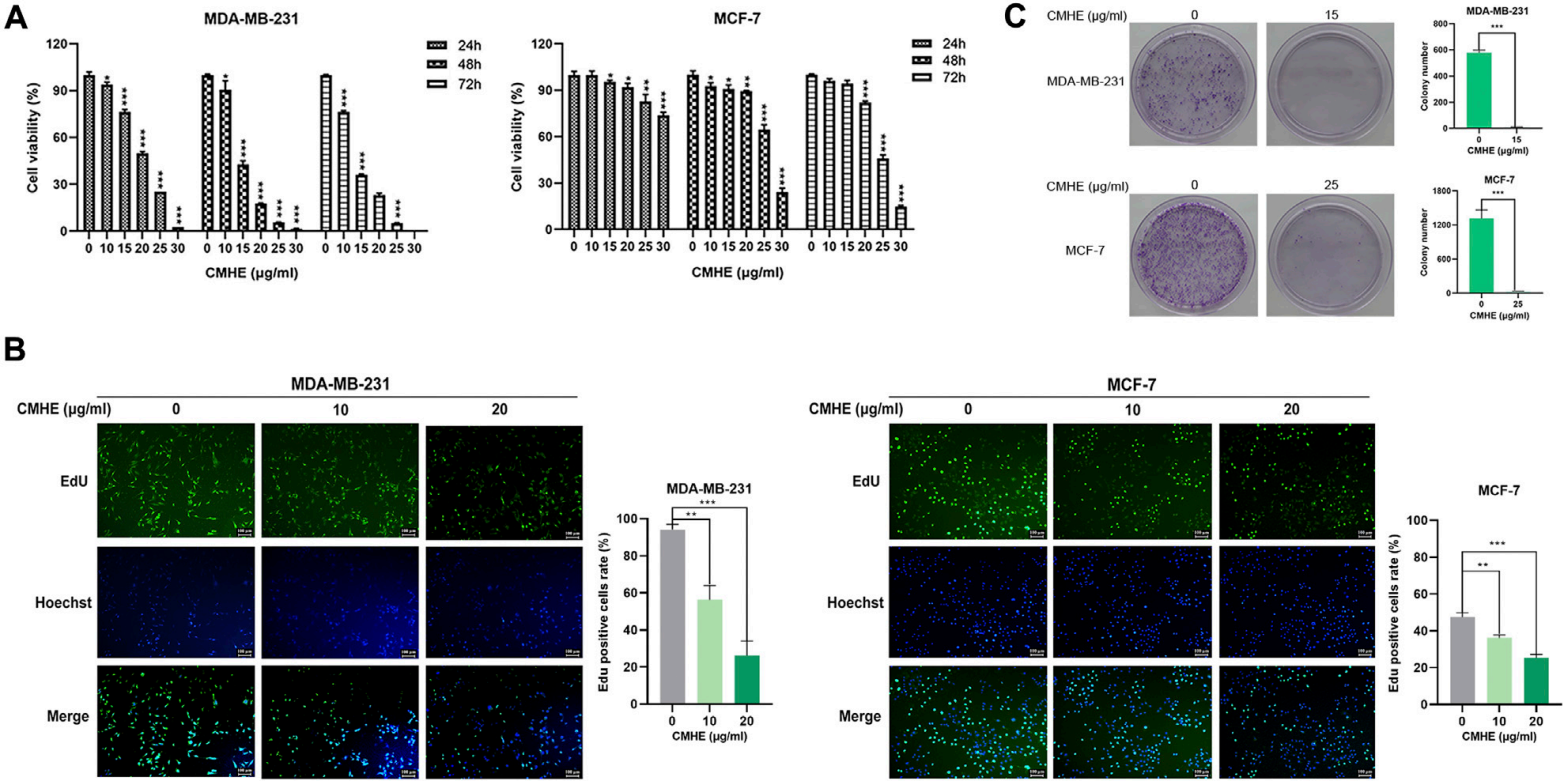
CMHE suppressed BC cell growth. **(A)** BC cell viability after CMHE treatment was detected using an MTT assay. **(B)** The proliferation of BC cells was explored by EdU cell proliferation assay after 48-h of CMHE treatment at 0, 10, and 20 μg/mL (100×). **(C)** The above two cells were exposed or unexposed to CMHE for 12 days before the colony formation assay. ^*^ *P* < 0.05, ^**^ *P* < 0.01, ^***^ *P* < 0.001.

In the present study, human BC MDA-MB-231 and MCF-7 cells were exposed to CMHE (0, 10, 15, 20, 25, and 30 μg/mL) for 1/2/3 days, separately. CMHE was found to significantly suppress BC cell viability, and the IC50 values of CMHE on MDA-MB-231 and MCF-7 cells for 48 h were 14.48 μg/mL and 26.50 μg/mL, respectively (Figure 1A). The effect of CMHE on the cell viability of human normal breast epithelial MCF-10A cells was also assessed (Supplementary Figure S2). EdU assay demonstrated a decrease in the bright green fluorescence in these two cells after CMHE treatment, indicating the suppression of BC cell proliferation by CMHE (Figure 1B). Moreover, CMHE dramatically repressed the colony formation capabilities of these two cells (Figure 1C) and markedly inhibited the growth of BC cells.

### 3.2 CMHE mediated arrest of the cell cycle at G0/G1 phase and promotion of apoptosis in human BC cells

**FIGURE 2:**
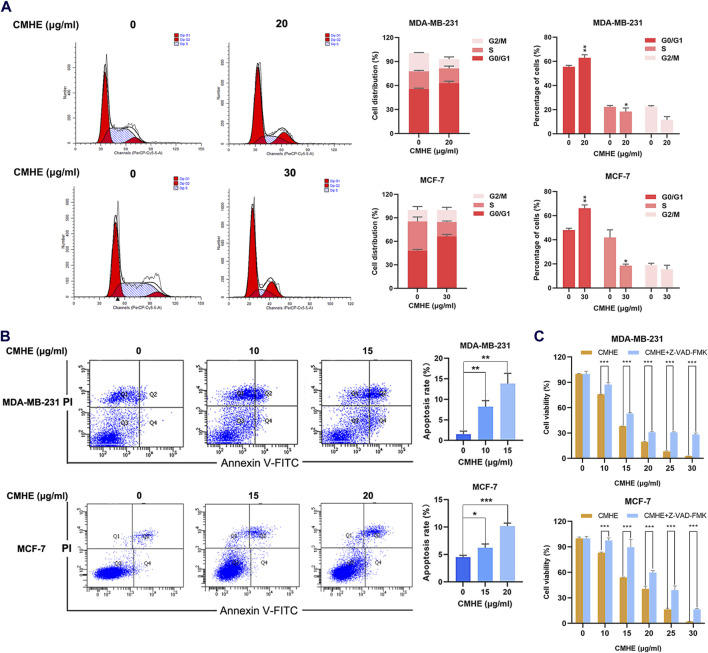
CMHE induced cell cycle arrest in the G0/G1 phase and promoted apoptotic cell death in human breast cancer cells. **(A)** BC cells subjected to 48-h of CMHE treatments were explored using flow cytometry to observe the cell cycle. **(B)** BC cells subjected to 48-h CMHE treatment were subjected to FCM to analyze apoptosis by Annexin V-FITC/PI staining. **(C)** The viability of BC cells subjected to 48-h CMHE treatment with or without Z-VAD-FMK (20 µM) was measured through MTT assay. ^*^ *P* < 0.05, ^**^ *P* < 0.01, ^***^ *P* < 0.001.

The ability of CMHE to influence apoptosis and cell cycle was evaluated using a flow cytometry (FCM) assay. According to FCM results, the administration of CMHE elevated the proportion of G0/G1 cells (Figure 2A) indicating the CMHE-induced arrest of the cell cycle at the G0/G1 phase. Moreover, the detection of apoptosis by flow cytometry revealed that CMHE enhanced apoptosis in a dose-dependent fashion (Figure 2B). Moreover, Z-VAD-FMK is a caspase inhibitor to block apoptosis (Pero et al., 2018). Inhibition of apoptosis by Z-VAD-FMK attenuated BC cell vulnerability to CMHE exposure (Figure 2C). CMHE was therefore found to mediate apoptosis in human BC cells.

### 3.3 CMHE suppressed metastatic potential of human breast cancer cells *in vitro*

**FIGURE 3:**
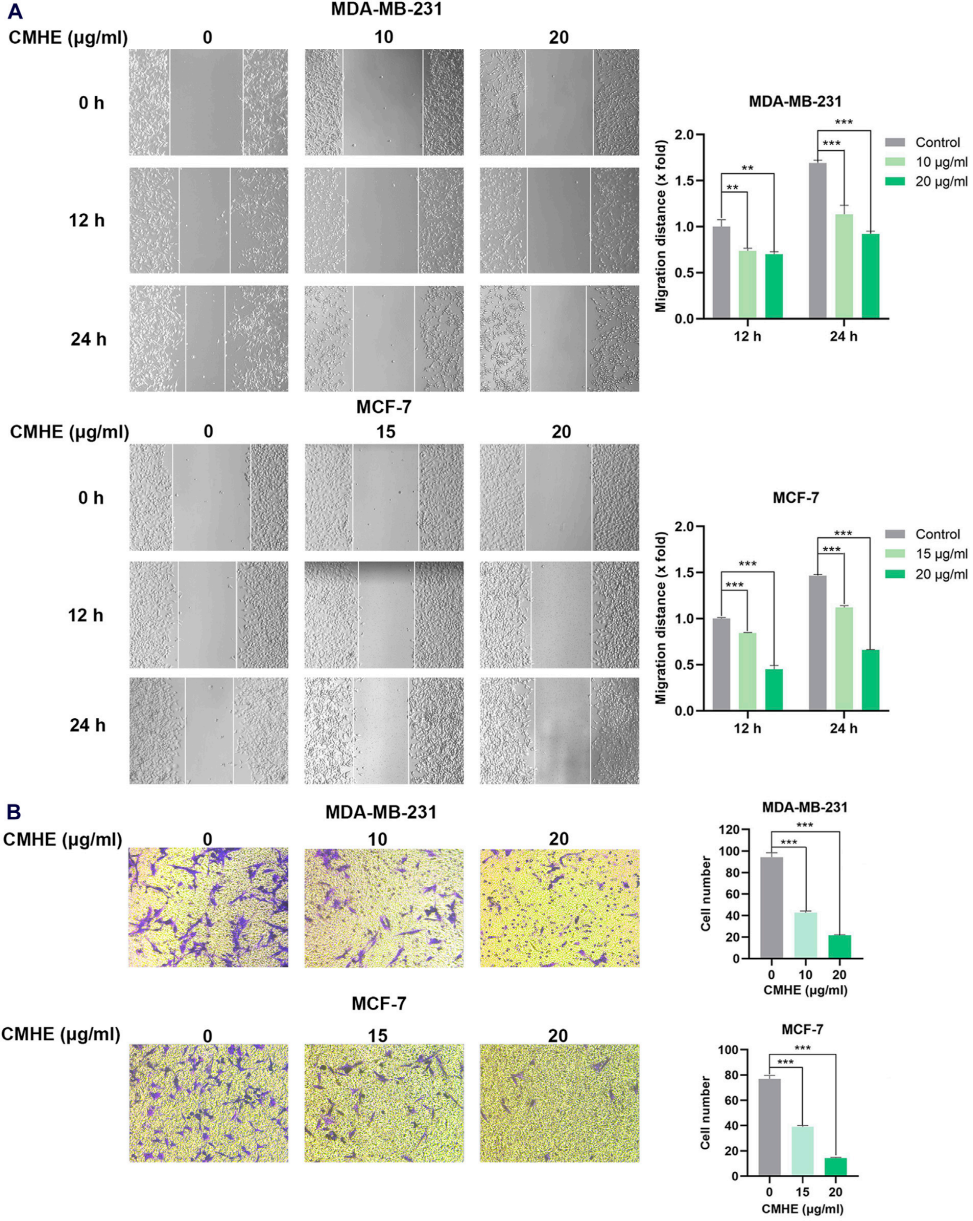
CMHE inhibited metastatic potential of human breast cancer cells *in vitro.* **(A)** BC cells subjected to CMHE treatment were used in a scratch assay. Typical images (100×, left panel); Quantitation (right panel). **(B)** BC cells subjected to 16- or 24-h of CMHE treatment were used for Matrigel invasion assay. Representative images (200×, left panel); Quantitative analysis (right panel). ^*^ *P* < 0.05, ^**^ *P* < 0.01, ^***^ *P* < 0.001.

The impact of CMHE on human BC cell migration and invasion capability was also investigated through wound-scratch assay and Matrigel invasion assay. As revealed by the scratch assay, CMHE treatment reduced the migration distance of BC cells, indicating that CMHE markedly inhibited the cells’ migration ability (Figure 3A). Additionally, cell number penetrating the Transwell membrane declined with CMHE, indicating the CMHE-induced repression of BC cell invasion (Figure 3B).

### 3.4 CMHE downregulated Cyclin D1/CDK4-Rb pathway within human BC cells

**FIGURE 4:**
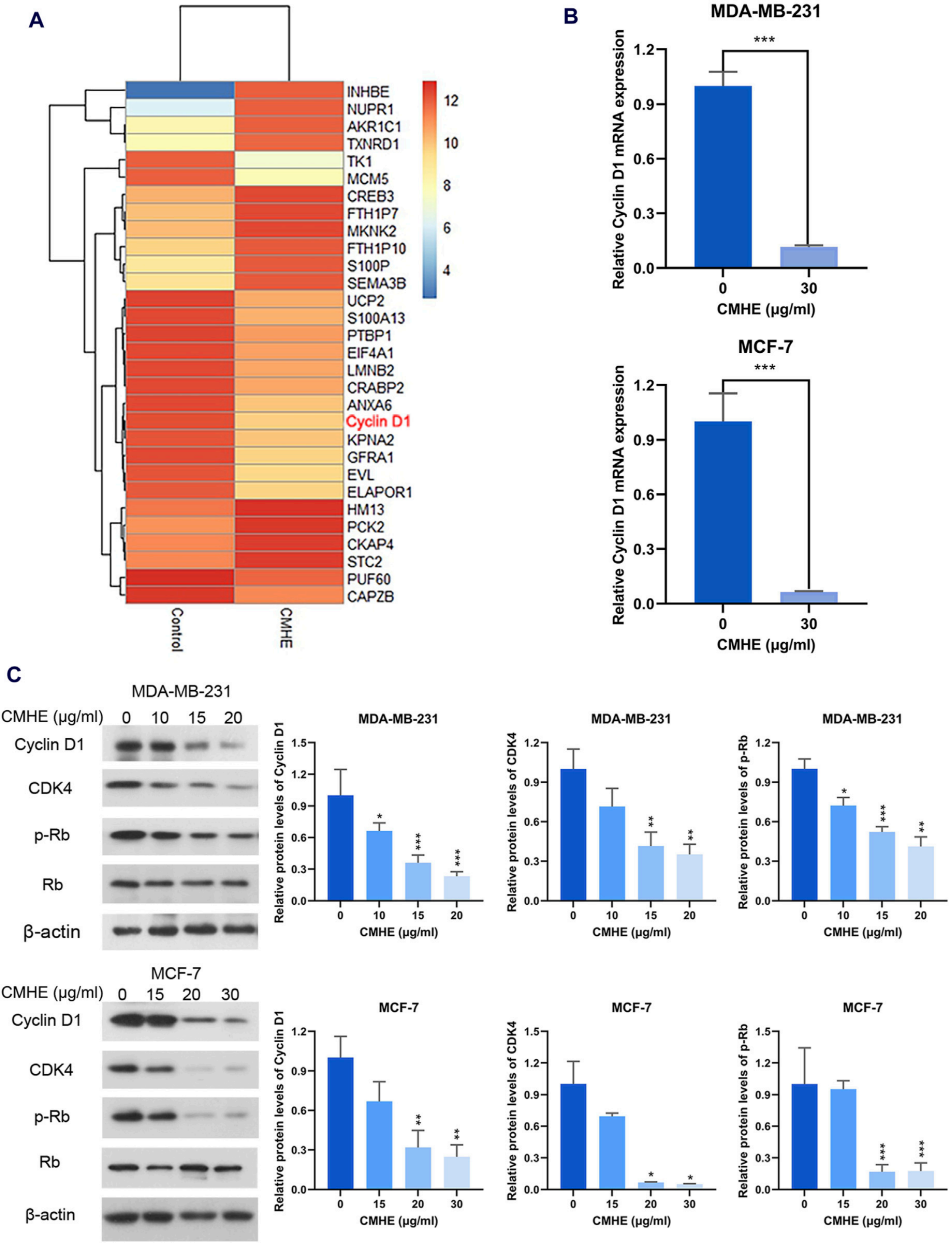
CMHE downregulated the Cyclin D1/CDK4-Rb pathway within human BC cells. **(A)** Heatmap showing differentially expressed genes (DEGs) in the MCF-7 cells after 48-h 30 μg/mL of CMHE treatment compared with non-treated cells. **(B)** QRT-PCR was carried out for determining Cyclin D1 mRNA expression within both the BC cell lines after 24-h of CMHE treatment at 30 μg/mL. **(C)** Immunoblot analysis of Cyclin D1/CDK4-Rb pathway within the BC cell lines after 48-h of CMHE treatment. ^*^ *P* < 0.05, ^**^ *P* < 0.01, ^***^ *P* < 0.001.

To investigate the anti-BC mechanism of CMHE, RNA sequencing analysis was performed for MCF-7 cells before and after CMHE treatment. RNA sequencing results revealed that Cyclin D1, a key gene in the G1 phase of the cell cycle, was downregulated within BC MCF-7 cells treated with CMHE (Figure 4A). Moreover, quantitative real-time PCR confirmed a significant decrease in Cyclin D1 mRNA expression in the BC cell lines after CMHE treatment (Figure 4B). Cyclin D1/CDK4-Rb signaling pathway has an important effect on the G1 phase in the cell cycle (Ding et al., 2020). Thus, the impact of CMHE on the Cyclin D1/CDK4-Rb cascade in human BC cells was explored. Immunoblotting analysis revealed a dramatic reduction in Cyclin D1 and CDK4 protein expression, and Rb phosphorylation after CMHE treatment (Figure 4C), indicating CMHE-induced inhibition of Cyclin D1/CDK4-Rb cascade in the human BC cells.

### 3.5 Cyclin D1/CDK4 signaling cascade participated in the anti-breast cancer effect of CMHE

**FIGURE 5:**
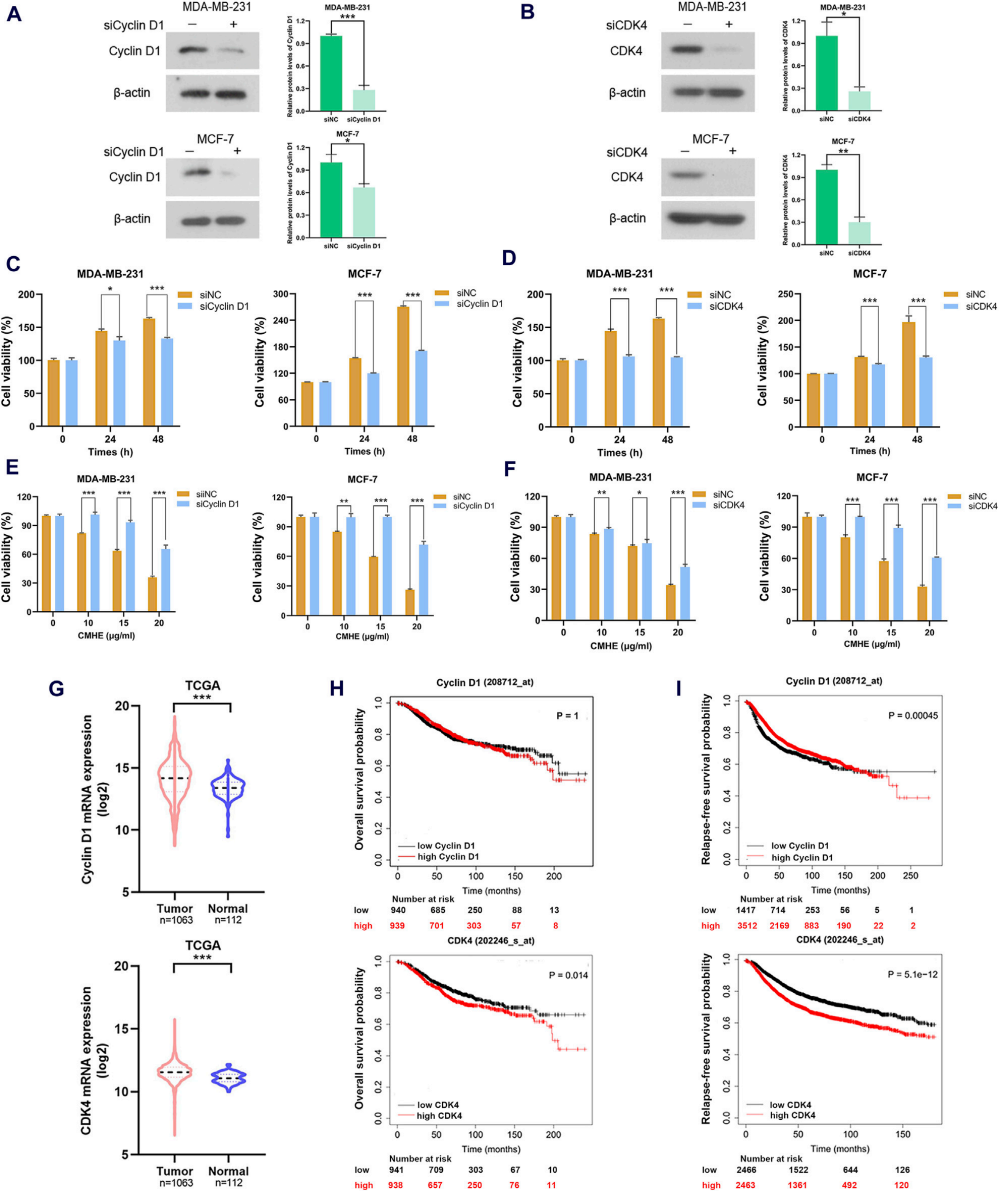
Cyclin D1/CDK4 signaling cascade participated in the anti-breast cancer effect of CMHE. **(A, B)** BC cells subjected to siRNAs targeting Cyclin D1 or CDK4 transfection were collected for immunoblotting analysis of Cyclin D1 and CDK4. **(C, D)** Detection of the proliferative ability of cells transfected with target gene siRNAs or negative control (NC) siRNAs was carried out using MTT assay. **(E, F)** The BC cell viability after target gene siRNAs or negative control (NC) siRNAs transfection in the presence of CMHE for 48 h was measured using an MTT assay. **(G)** Cyclin D1 and CDK4 levels within BC and non-BC tissues were analyzed through the TCGA database. **(H, I)** Kaplan-Meier (KM) plotter analysis of OS and RFS in BC cases based on Cyclin D1 or CDK4 expression (http://kmplot.com). ^*^ *P* < 0.05, ^**^ *P* < 0.01, ^***^ *P* < 0.001.

With the aim of investigating the involvement of the Cyclin D1/CDK4 signaling cascade in the growth suppression of human BC cells induced by CMHE, an RNA interference assay was carried out to knock down Cyclin D1 or CDK4 levels within two BC cell lines. Cyclin D1 expression was found to be knocked down by siRNAs in these two cells (Figure 5A). In addition, CDK4 expression was silenced by siRNAs (Figure 5B) and the depletion of Cyclin D1 or CDK4 expression suppressed the proliferation of these two cells (Figures 5C, D). Most importantly, the reduction of Cyclin D1 or CDK4 expression impaired the vulnerability of BC cells to CMHE treatment (Figures 5E, F). Besides, Cyclin D1 and CDK4 expression within tissues from BC patients was assessed by analysing data from the publicly available The Cancer Genome Atlas (TCGA) database. Cyclin D1 and CDK4 levels were found to be increased in the BC tissues in relative to controls (Figure 5G). The Kaplan-Meier plotter analysis of a publicly available database (http://kmplot.com) (Gyorffy, 2021; Lanczky and Gyorffy, 2021) also showed that high Cyclin D1 or CDK4 expression predicted dismal overall survival (OS) as well as relapse-free survival (RFS) in BC cases (Figures 5H, I). Therefore, the downregulation of the Cyclin D1/CDK4 signaling cascade was identified as the mechanism responsible for the anti-BC effect of CMHE.

### 3.6 CMHE suppressed tumor development in the BC mouse model

**FIGURE 6:**
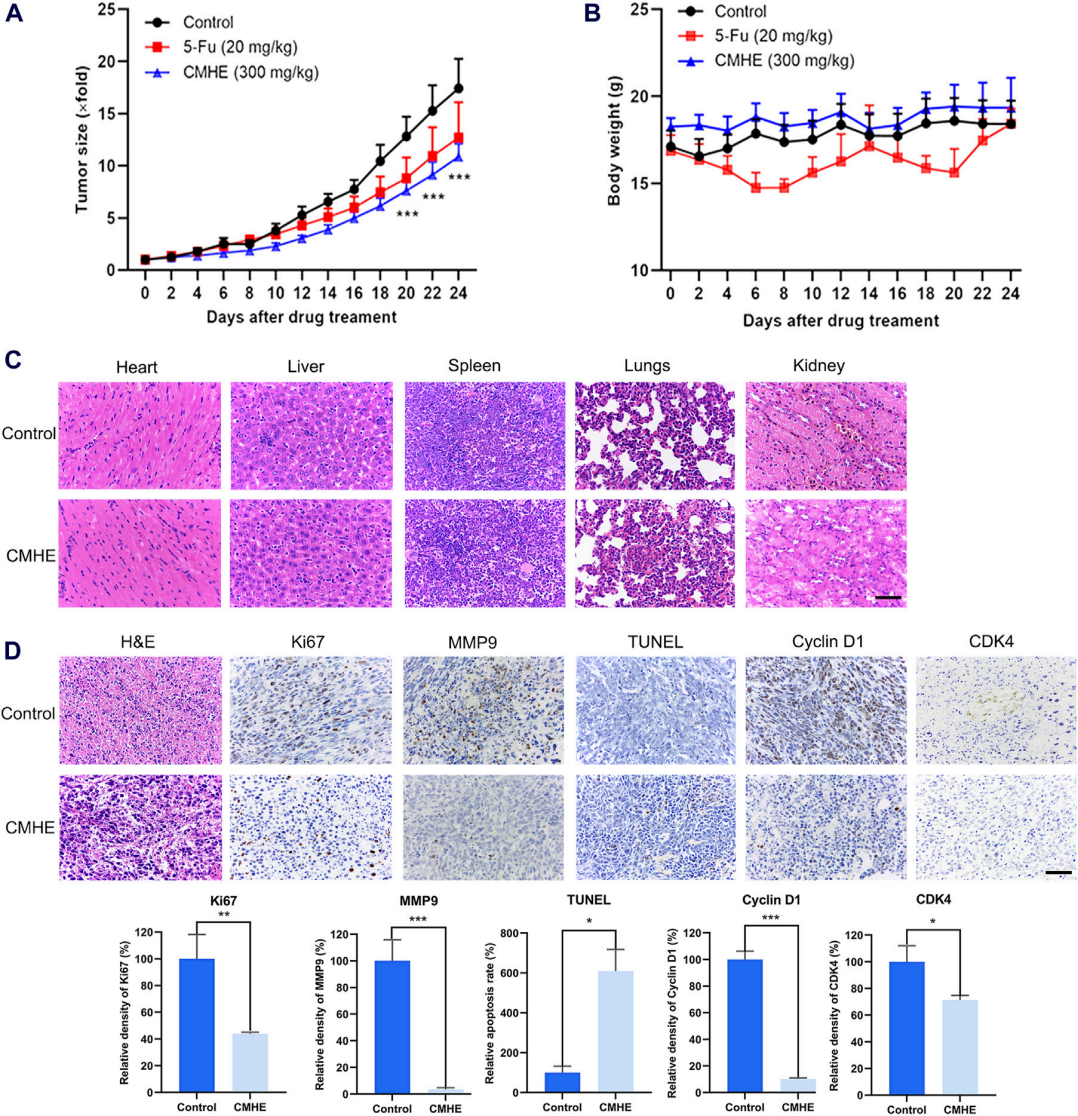
CMHE blunted tumor growth in 4T1 breast cancer-bearing mice. **(A)** Tumor volume relative growth curves and **(B)** body weight curves of 4T1 BC-bearing mice after treatment with PBS, CMHE (300 mg/kg), or 5-Fu (20 mg/kg). **(C)** Major organs of mice were subjected to H&E staining. **(D)** Tumor tissues of tumor-bearing mice were subjected to immunohistochemistry staining, and quantification was carried out with the application of Image-Pro Plus 6.0 software. Scale bar = 50 µm ^*^ *P* < 0.05, ^**^ *P* < 0.01, ^***^ *P* < 0.001.

For investigating the anti-BC impact of CMHE *in vivo,* the 4T1 breast cancer-bearing mouse model was established. The administration of CMHE markedly restrained tumorigenesis in 4T1 BC-bearing mice, with an approximate tumor inhibition rate of 37.66% (Figure 6A). Moreover, CMHE administration led to no significant effect on the weight of the mouse (Figure 6B). Furthermore, H&E staining analysis revealed no distinct toxic effect on mouse liver, heart, lung, spleen, and kidney after CMHE treatment (Figure 6C). Additionally, the immunohistochemical analysis indicated reduced expression of Ki67, MMP9, Cyclin D1, and CDK4 in the tumor tissues of CMHE-treated mice (Figure 6D), implying the repression of the proliferative and metastatic ability of cancer cells in the tumor tissues by CMHE treatment. TUNEL staining revealed the facilitation of apoptosis in the tumor tissues after CMHE treatment (Figure 6D).

## 4 Discussion

Recently, studying the anti-BC effect of TCM has been receiving increasing attention from researchers worldwide (Cohen et al., 2002; Porter et al., 2017; Lee et al., 2020). *Commiphora myrrha* is an important component of the Xihuang Pill widely used to treat BC in China. However, the anti-BC effect and the underlying mechanism of action of *C. myrrha* is largely unknown. This study demonstrated that the treatment of CMHE led to the suppression of the development of human BC cells by inducing cell cycle arrest at G0/G1 and apoptotic cell death via the suppression of the Cyclin D1/CDK4-Rb pathway, generating the anti-BC activity of CMHE.

The abnormal proliferation of cells is closely associated with the progression of cancer (Beresford et al., 2006). The immunohistochemical determination for Ki67 has been widely used as a cell proliferation biomarker of BC (Nielsen et al., 2021). In this study, the treatment of CMHE dramatically hindered the growth of human BC cells *in vitro,* and markedly decreased levels of Ki67 in tumor tissues. Cell cycle dysregulation is one of the major reasons behind the aberrant proliferation of cancer cells (Matthews et al., 2022). Quercetin exerted anti-BC activity by inducing G1 phase arrest (Ranganathan et al., 2015). This study demonstrated the ability of CMHE to trigger cell cycle arrest at the G0/G1 phase in BC cells. Moreover, apoptosis is a critical target in cancer treatment (Pistritto et al., 2016). Activation of apoptosis is crucial to the tumor regression as a response to chemotherapy, and the effectiveness of chemotherapeutic drugs has been connected to their ability to induce apoptosis (Pfeffer and Singh, 2018; Srivastava and Srivastava, 2019). FCM, pharmacological inhibition, and TUNEL staining of tumor tissues revealed the promotion of apoptotic cell death of BC cells by CMHE. Additionally, CMHE administration significantly blocked tumor growth in mice. Collectively, CMHE exhibited promising anti-BC effects *in vitro* and *in vivo*.

Most of the cases associated with BC can be related to metastasis. Thus, preventing or reducing tumor cell metastasis is a critical strategy for treating BC in patients (Maroufi et al., 2020). Matrix metalloproteinases (MMPs) belong to the extracellular matrix-degrading enzyme family (Wu et al., 2021). MMP9 promotes metastasis of cancer cells by the degradation of the extracellular matrix and basement membrane (Allen et al., 2022), and the levels of MMP9 are positively related to the rate of distant metastasis (Owyong et al., 2019). This investigation showed that CMHE treatment led to the inhibition of metastasis in human BC cells *in vitro*. Furthermore, immunohistochemical analysis revealed a decrease in the expression of MMP9 in the tumor tissues of CMHE-treated mice. CMHE may therefore be an anti-BC candidate drug with anti-metastatic potential.

Cyclin D1/CDK4-Rb pathway has a significant role in the G1 phase, which is implicated in the development of cancer (Gao et al., 2018; Martinez-Alonso and Malumbres, 2020). Cyclin D1-CDK4 interaction is vital during the G1 phase and is involved in cell division, manipulating the phosphorylation of Rb to activate downstream genes (Kusume et al., 1999; Pan et al., 2001; Sobhani et al., 2019). Studies have reported that the downregulation of the Cyclin D1/CDK4-Rb pathway suppresses cell proliferation and promotes apoptosis of cancer cells. Kaempferol has been shown to hinder cell growth and induce apoptosis in human gallbladder cancer cells via Cyclin D1/CDK4 signaling pathway (Liu et al., 2021). Aspirin induced human colorectal cancer (CRC) cell apoptosis by suppressing Cyclin D1/CDK4 pathway (Thoms et al., 2007). Additionally, Cyclin D1/CDK4-Rb pathway regulates the metastatic capacity of cancer cells. Dihydroartemisin downregulated PCNA and MMP-2, mediated by p16-Cyclin D1/CDK4-Rb pathway, suppresses the cell growth and metastasis of human gastric cancer (GC) cells (Fan et al., 2020). Aqueous extract obtained from *Scutellaria baicalensis* resulted in a reduction in the expression of MMP-2 by downregulating Cyclin D1, thus hindering the growth and invasion of tumor cells (Park et al., 2011). Results from RNA-sequencing analysis, qRT-PCR, immunoblotting, and immunohistochemical staining revealed that CMHE inhibited Cyclin D1/CDK4-Rb pathway in BC cells. Moreover, the depletion of Cyclin D1 or CDK4 impaired the vulnerability of human BC cells to CMHE treatment. Additionally, database analysis (http://kmplot.com) showed that high Cyclin D1 or CDK4 levels predicted poor OS and RFS in BC patients. Collectively, the downregulation of the Cyclin D1/CDK4-Rb signaling cascade was responsible for the anti-BC effect of CMHE.

## 5 Conclusion

In summary, CMHE exhibited promising anti-BC effects *in vitro* and *in vivo.* CMHE suppressed the growth of human BC cells through triggering cell cycle arrest at G0/G1 and apoptotic cell death by inhibiting the Cyclin D1/CDK4-Rb pathway, generating the anti-BC effect of CMHE. Therefore, CMHE may be a potential drug candidate for BC therapy, particularly for those harboring aberrant Cyclin D1/CDK4-Rb pathway activation.

## Data Availability

The original contributions presented in the study are included in the article/Supplementary Material, further inquiries can be directed to the corresponding author/s.

## Abbreviations

TCM, traditional Chinese medicine; CMHE, Commiphora myrrha n-hexane extract; IHC analysis, immunohistochemical analysis; RPMI-1640, Roswell Park Memorial Institute 1640 medium; DMEM, Dulbecco’s Modified Eagle Medium; FBS, fetal bovine serum; TCGA, The Cancer Genome Atlas; OS, overall survival; RFS, relapse-free survival; MMPs, matrix metalloproteinases.

## References

[B1] Accordino M. K. Wright J. D. Vasan S. Neugut A. I. Hillyer G. C. Hu J. C. (2016). Use and costs of disease monitoring in women with metastatic breast cancer. J. Clin. Oncol. 34 (24), 2820–2826. 10.1200/JCO.2016.66.6313 27161970 PMC5012664

[B2] Allen J. L. Hames R. A. Mastroianni N. M. Greenstein A. E. Weed S. A. (2022). Evaluation of the matrix metalloproteinase 9 (MMP9) inhibitor Andecaliximab as an Anti-invasive therapeutic in Head and neck squamous cell carcinoma. Oral Oncol. 132, 106008. 10.1016/j.oraloncology.2022.106008 35803110 PMC13524229

[B3] Beresford M. J. Wilson G. D. Makris A. (2006). Measuring proliferation in breast cancer: practicalities and applications. Breast Cancer Res. 8 (6), 216. 10.1186/bcr1618 17164010 PMC1797032

[B4] Cohen I. Tagliaferri M. Tripathy D. (2002). Traditional Chinese medicine in the treatment of breast cancer. Semin. Oncol. 29 (6), 563–574. 10.1053/sonc.2002.50005 12516039

[B5] Ding L. Cao J. Lin W. Chen H. Xiong X. Ao H. (2020). The roles of cyclin-dependent kinases in cell-cycle progression and therapeutic strategies in human breast cancer. Int. J. Mol. Sci. 21 (6), 1960. 10.3390/ijms21061960 32183020 PMC7139603

[B6] Fan H. N. Zhu M. Y. Peng S. Q. Zhu J. S. Zhang J. Qu G. Q. (2020). Dihydroartemisinin inhibits the growth and invasion of gastric cancer cells by regulating cyclin D1-CDK4-Rb signaling. Pathol. Res. Pract. 216 (2), 152795. 10.1016/j.prp.2019.152795 31879047

[B7] Gao A. Sun T. Ma G. Cao J. Hu Q. Chen L. (2018). LEM4 confers tamoxifen resistance to breast cancer cells by activating cyclin D-CDK4/6-Rb and ERα pathway. Nat. Commun. 9 (1), 4180. 10.1038/s41467-018-06309-8 30301939 PMC6177406

[B8] Ge A. Yang K. Deng X. Zhao D. Ge J. Liu L. (2022). The efficacy and safety of Xihuang Pill/capsule in adjuvant treatment of breast cancer: a systematic review and meta-analysis of 26 randomized controlled trials. J. Ethnopharmacol. 295, 115357. 10.1016/j.jep.2022.115357 35545184

[B9] Gyorffy B. (2021). Survival analysis across the entire transcriptome identifies biomarkers with the highest prognostic power in breast cancer. Comput. Struct. Biotechnol. J. 19, 4101–4109. 10.1016/j.csbj.2021.07.014 34527184 PMC8339292

[B10] Hortobagyi G. N. Van Poznak C. Harker W. G. Gradishar W. J. Chew H. Dakhil S. R. (2017). Continued treatment effect of zoledronic acid dosing every 12 vs 4 Weeks in women with breast cancer metastatic to bone: the OPTIMIZE-2 randomized clinical trial. JAMA Oncol. 3 (7), 906–912. 10.1001/jamaoncol.2016.6316 28125763 PMC5824238

[B11] Hu Z. Wang Y. Huang F. Chen R. Li C. Wang F. (2015). Brain-expressed X-linked 2 is pivotal for hyperactive mechanistic target of rapamycin (mTOR)-mediated tumorigenesis. J. Biol. Chem. 290 (42), 25756–25765. 10.1074/jbc.M115.665208 26296882 PMC4646217

[B12] Hu Z. Yang A. Su G. Zhao Y. Wang Y. Chai X. (2016). Huaier restrains proliferative and invasive potential of human hepatoma SKHEP-1 cells partially through decreased Lamin B1 and elevated NOV. Sci. Rep. 6, 31298. 10.1038/srep31298 27503760 PMC4977525

[B13] Hung Y. L. Leung S. S. Chiu S. P. Li P. Y. Kan A. C. Lo C. C. (2022). Perceptions about traditional Chinese medicine use among Chinese breast cancer survivors: a qualitative study. Cancer Med. 12 (2), 1997–2007. 10.1002/cam4.5046 36073533 PMC9883569

[B43] Jin F. Jiang K. Ji S. Wang L. Ni Z. Huang F. (2017). Deficient TSC1/TSC2-complex suppression of SOX9-osteopontin-AKT signalling cascade constrains tumour growth in tuberous sclerosis complex. Hum. Mol. Genet. 26 (2), 407–419. 10.1093/hmg/ddw397 28013293

[B14] Kusume T. Tsuda H. Kawabata M. Inoue T. Umesaki N. Suzuki T. (1999). The p16-cyclin D1/CDK4-pRb pathway and clinical outcome in epithelial ovarian cancer. Clin. Cancer Res. 5 (12), 4152–4157. 10632354

[B15] Lanczky A. Gyorffy B. (2021). Web-based survival analysis tool tailored for medical research (KMplot): development and implementation. J. Med. Internet Res. 23 (7), e27633. 10.2196/27633 34309564 PMC8367126

[B16] Lee Y. C. Chen Y. H. Huang Y. C. Lee Y. F. Tsai M. Y. (2020). Effectiveness of combined treatment with traditional Chinese medicine and western medicine on the prognosis of patients with breast cancer. J. Altern. Complement. Med. 26 (9), 833–840. 10.1089/acm.2019.0200 32924556

[B17] Liu Z. Q. Yao G. L. Zhai J. M. Hu D. W. Fan Y. G. (2021). Kaempferol suppresses proliferation and induces apoptosis and DNA damage in human gallbladder cancer cells through the CDK4/CDK6/cyclin D1 pathway. Eur. Rev. Med. Pharmacol. Sci. 25 (3), 1311–1321. 10.26355/eurrev_202102_24836 33629301

[B18] Madia V. N. De Angelis M. De Vita D. Messore A. De Leo A. Ialongo D. (2021). Investigation of Commiphora myrrha (nees) Engl. Oil and its main components for antiviral activity. Pharm. (Basel) 14 (3), 243. 10.3390/ph14030243 PMC7999460 33803165

[B19] Maroufi N. F. Ashouri N. Mortezania Z. Ashoori Z. Vahedian V. Amirzadeh-Iranaq M. T. (2020). The potential therapeutic effects of melatonin on breast cancer: an invasion and metastasis inhibitor. Pathol. Res. Pract. 216 (10), 153226. 10.1016/j.prp.2020.153226 32987338

[B20] Martinez-Alonso D. Malumbres M. (2020). Mammalian cell cycle cyclins. Semin. Cell Dev. Biol. 107, 28–35. 10.1016/j.semcdb.2020.03.009 32334991

[B21] Matthews H. K. Bertoli C. De Bruin R. a. M. (2022). Cell cycle control in cancer. Nat. Rev. Mol. Cell Biol. 23 (1), 74–88. 10.1038/s41580-021-00404-3 34508254

[B22] Nielsen T. O. Leung S. C. Y. Rimm D. L. Dodson A. Acs B. Badve S. (2021). Assessment of Ki67 in breast cancer: updated recommendations from the international Ki67 in breast cancer working group. J. Natl. Cancer Inst. 113 (7), 808–819. 10.1093/jnci/djaa201 33369635 PMC8487652

[B23] Owyong M. Chou J. Van Den Bijgaart R. J. Kong N. Efe G. Maynard C. (2019). MMP9 modulates the metastatic cascade and immune landscape for breast cancer anti-metastatic therapy. Life Sci. Alliance 2 (6), e201800226. 10.26508/lsa.201800226 31727800 PMC6856766

[B24] Pan W. Cox S. Hoess R. H. Grafstrom R. H. (2001). A cyclin D1/cyclin-dependent kinase 4 binding site within the C domain of the retinoblastoma protein. Cancer Res. 61 (7), 2885–2891. 11306463

[B25] Park K. I. Park H. S. Kang S. R. Nagappan A. Lee D. H. Kim J. A. (2011). Korean Scutellaria baicalensis water extract inhibits cell cycle G1/S transition by suppressing cyclin D1 expression and matrix-metalloproteinase-2 activity in human lung cancer cells. J. Ethnopharmacol. 133 (2), 634–641. 10.1016/j.jep.2010.10.057 21073943

[B26] Peng L. Zhang K. Li Y. Chen L. Gao H. Chen H. (2022). Real-world evidence of traditional Chinese medicine (TCM) treatment on cancer: a literature-based review. Evid. Based Complement. Altern. Med. 2022, 7770380. 10.1155/2022/7770380 PMC9259235 35815277

[B27] Pero M. E. Zullo G. Esposito L. Iannuzzi A. Lombardi P. De Canditiis C. (2018). Inhibition of apoptosis by caspase inhibitor Z-VAD-FMK improves cryotolerance of *in vitro* derived bovine embryos. Theriogenology 108, 127–135. 10.1016/j.theriogenology.2017.11.031 29207293

[B28] Pfeffer C. M. Singh A. T. K. (2018). Apoptosis: a target for anticancer therapy. Int. J. Mol. Sci. 19 (2), 448. 10.3390/ijms19020448 29393886 PMC5855670

[B29] Pistritto G. Trisciuoglio D. Ceci C. Garufi A. D'orazi G. (2016). Apoptosis as anticancer mechanism: function and dysfunction of its modulators and targeted therapeutic strategies. Aging (Albany NY) 8 (4), 603–619. 10.18632/aging.100934 27019364 PMC4925817

[B30] Porter D. Cochrane S. Zhu X. (2017). Current usage of traditional Chinese medicine for breast cancer-A narrative approach to the experiences of women with breast cancer in Australia-A pilot study. Med. (Basel) 4 (2), 20. 10.3390/medicines4020020 PMC5590056 28930235

[B31] Ranganathan S. Halagowder D. Sivasithambaram N. D. (2015). Quercetin suppresses twist to induce apoptosis in MCF-7 breast cancer cells. PLoS One 10 (10), e0141370. 10.1371/journal.pone.0141370 26491966 PMC4619597

[B32] Shen T. Li G. H. Wang X. N. Lou H. X. (2012). The genus Commiphora: a review of its traditional uses, phytochemistry and pharmacology. J. Ethnopharmacol. 142 (2), 319–330. 10.1016/j.jep.2012.05.025 22626923

[B33] Sobhani N. D'angelo A. Pittacolo M. Roviello G. Miccoli A. Corona S. P. (2019). Updates on the CDK4/6 inhibitory strategy and combinations in breast cancer. Cells 8 (4), 321. 10.3390/cells8040321 30959874 PMC6523967

[B34] Srivastava N. S. Srivastava R. a. K. (2019). Curcumin and quercetin synergistically inhibit cancer cell proliferation in multiple cancer cells and modulate Wnt/β-catenin signaling and apoptotic pathways in A375 cells. Phytomedicine 52, 117–128. 10.1016/j.phymed.2018.09.224 30599890

[B35] Sun Q. Chen X. Ma J. Peng H. Wang F. Zha X. (2011). Mammalian target of rapamycin up-regulation of pyruvate kinase isoenzyme type M2 is critical for aerobic glycolysis and tumor growth. Proc. Natl. Acad. Sci. U. S. A. 108 (10), 4129–4134. 10.1073/pnas.1014769108 21325052 PMC3054028

[B36] Tarantino P. Corti C. Schmid P. Cortes J. Mittendorf E. A. Rugo H. (2022). Immunotherapy for early triple negative breast cancer: research agenda for the next decade. NPJ Breast Cancer 8 (1), 23. 10.1038/s41523-022-00386-1 35181659 PMC8857212

[B37] Thoms H. C. Dunlop M. G. Stark L. A. (2007). p38-mediated inactivation of cyclin D1/cyclin-dependent kinase 4 stimulates nucleolar translocation of RelA and apoptosis in colorectal cancer cells. Cancer Res. 67 (4), 1660–1669. 10.1158/0008-5472.CAN-06-1038 17308107

[B38] Wang C. C. Liang N. Y. Xia H. Wang R. Y. Zhang Y. F. Huo H. X. (2022). Cytotoxic sesquiterpenoid dimers from the resin of Commiphora myrrha Engl. Phytochemistry 204, 113443. 10.1016/j.phytochem.2022.113443 36169037

[B39] Wu H. T. Lin J. Liu Y. E. Chen H. F. Hsu K. W. Lin S. H. (2021). Luteolin suppresses androgen receptor-positive triple-negative breast cancer cell proliferation and metastasis by epigenetic regulation of MMP9 expression via the AKT/mTOR signaling pathway. Phytomedicine 81, 153437. 10.1016/j.phymed.2020.153437 33352494

[B40] Wu J. Luo D. Li S. (2020). Network pharmacology-oriented identification of key proteins and signaling pathways targeted by Xihuang Pill in the treatment of breast cancer. Breast Cancer (Dove Med. Press) 12, 267–277. 10.2147/BCTT.S284076 33324095 PMC7733446

[B41] Xu H. B. Chen X. Z. Yu Z. L. Xue F. (2023). Guggulsterone from Commiphora mukul potentiates anti-glioblastoma efficacy of temozolomide *in vitro* and *in vivo* via down-regulating EGFR/PI3K/Akt signaling and NF-κB activation. J. Ethnopharmacol. 301, 115855. 10.1016/j.jep.2022.115855 36280019

[B42] Yang A. Fan H. Zhao Y. Chen X. Zhu Z. Zha X. (2019). An immune-stimulating proteoglycan from the medicinal mushroom Huaier up-regulates NF-κB and MAPK signaling via Toll-like receptor 4. J. Biol. Chem. 294 (8), 2628–2641. 10.1074/jbc.RA118.005477 30602571 PMC6393594

