# Supplementary material for: Commiphora myrrha n-hexane extract suppressed breast cancer progression through induction of G0/G1 phase arrest and apoptotic cell death by inhibiting the Cyclin D1/CDK4-Rb signaling pathway
Source: Front Pharmacol. 2024 Aug 5;15:1425157. doi: 10.3389/fphar.2024.1425157 (PMC11330881; doi:10.3389/fphar.2024.1425157)
Supplement: Supplementary file 1 [file DataSheet1.docx]

A


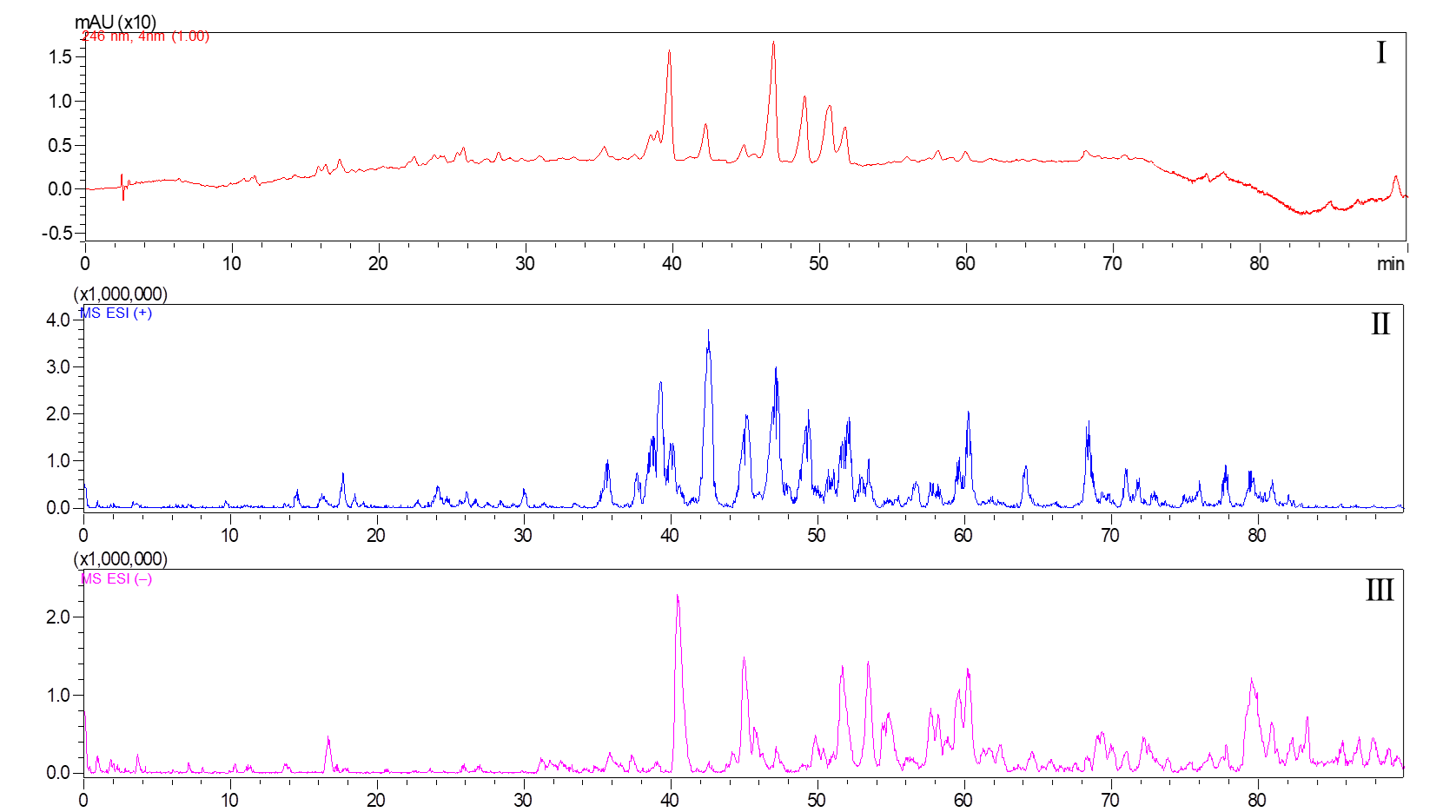


B

**Supplementary Figure S1.** HPLC-MS-IT-TOF total ion chromatogram spectrum (A) and ^1^H-NMR spectrum (B) of CMHE. The chromatographic separation was performed on an Agilent Zorbax SB C18 column (4.6 × 250 mm, 5 μm, Waters Technologies, USA). Acetonitrile (A)-0.1% aqueous with formic acid (B) were used as the mobile phase for HPLC-MS-IT-TOF analysis. The elution condition was applied with a gradient program as follows: 0-50 min, 30%-60% A; 50-65 min, 60%-70% A; 65-75 min, 70%-85% A; 75-80 min, 85%-95% A; 80-90 min, 95% A. Aliquots of 10 μL were injected into HPLC-MS-IT-TOF system for analysis.


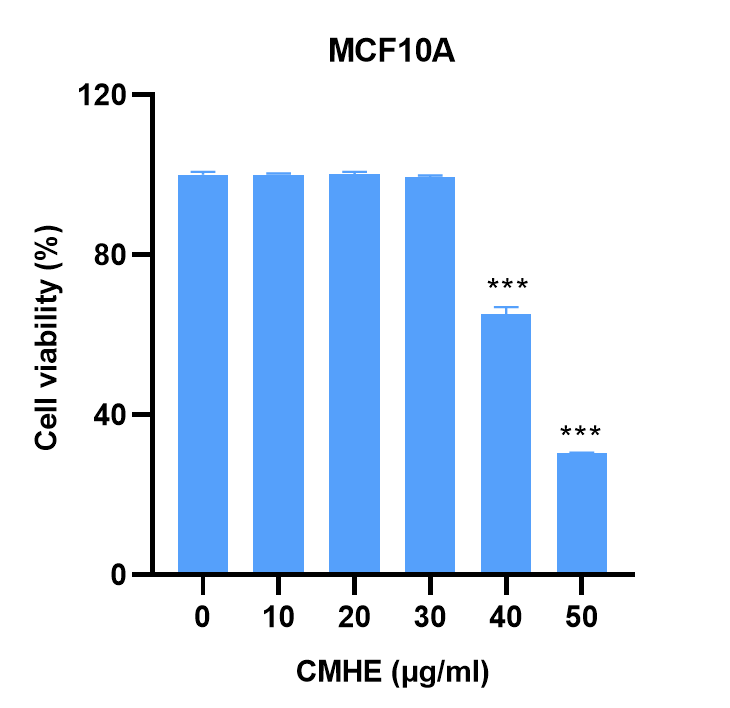


**Supplementary Figure S2.** The cell viability of human normal breast epithelial MCF10A cells after CMHE treatment was detected using an MTT assay. ^***^*P* < 0.001.
